# Unlocking multiphoton emission from a single-photon source through mean-field engineering

**DOI:** 10.1126/sciadv.adw3395

**Published:** 2025-10-29

**Authors:** Sang Kyu Kim, Eduardo Zubizarreta Casalengua, Katarina Boos, Friedrich Sbresny, Carolin Calcagno, Hubert Riedl, Jonathan J. Finley, Carlos Antón-Solanas, Fabrice P. Laussy, Kai Müller, Lukas Hanschke, Elena del Valle

**Affiliations:** ^1^ Walter Schottky Institut, TUM School of Computation, Information and Technology, and MCQST, Technische Universität München, 85748 Garching, Germany.; ^2^ Institute for Advanced Study, Technische Universität München, 85748 Garching, Germany.; ^3^ Walter Schottky Institut, TUM School of Natural Sciences, and MCQST, Technische Universität München, 85748 Garching, Germany.; ^4^ Departamento de Física de Materiales, Instituto Nicolás Cabrera, Universidad Autónoma de Madrid, 28049 Madrid, Spain.; ^5^ Condensed Matter Physics Center (IFIMAC), Universidad Autónoma de Madrid, 28049 Madrid, Spain.; ^6^ Instituto de Ciencia de Materiales de Madrid ICMM-CSIC, 28049 Madrid, Spain.; ^7^ Departamento de Física Teórica de la Materia Condensada, Universidad Autónoma de Madrid, 28049 Madrid, Spain.

## Abstract

In the single-photon emission from a two-level system, multiphotons are generally regarded as accidental, undesired, and unrelated to the mechanism. In coherently driven systems, however, they form the cornerstone of single-photon emission, which arises from quantum interferences between virtual multiphoton fluctuations of the emitter and the Poissonian superposition of all number states induced by the driving. Here, we demonstrate how one can control the multiphoton dynamics by disrupting these quantum interferences through an external homodyne control of the emitter’s mean field. Experimentally, we observed a transition from single-photon to multiphoton emission, up to three-photon correlations. We show that, counterintuitively, quantum fluctuations always play a major qualitative role, even and, in fact, especially when their quantitative contribution is vanishing. Our findings provide distinct insights into the paradoxical character of quantum mechanics and open pathways for mean-field engineering as a tool for precision multiphoton control.

## Teaser

Disrupting quantum interferences with a laser reveals virtual multiphotons from a single-photon source.

## INTRODUCTION

Resonance fluorescence—the photoluminescence of a two-level system (TLS) at the same energy at which it is coherently driven—has been a hallmark of the quantum theory of light-matter interaction since its early days (*1*). This process has been extensively studied across various platforms, including atoms (*2*), superconducting qubits (*3*), molecules (*4*), ions (*5*), chiral artificial atoms (*6*), and solid-state quantum dots (*7*). The rich physics of resonance fluorescence has been investigated through a prolific literature over decades, ranging from multiphoton scattering (*8*) to fluorescence from a squeezed vacuum (*9*, *10*), passing by interferences between past and future quantum states (*11*) as well as quantum dynamical (*12*, *13*) and nonlocal (*14*) aspects. Among these phenomena, the generation of single photons has received notable attention as a promising resource for quantum technologies.

In quantum mechanics, any observed outcome arises from the probability distribution over all possible states. Single-photon emission of a TLS under weak coherent driving can be thus described by a quantum superposition of all photon number states, where multiphotons remain virtual. By “virtual,” we mean that their probability amplitudes play a role but cancel out in the measurement. Recently, the multiphoton aspect has been revisited for its spectacular manifestations of counterintuitive features of quantum physics. Prominently, Masters *et al.* (*15*) have shown how a single TLS can simultaneously emit two photons despite having only one transition available to do so. Liu *et al.* (*12*) have subsequently demonstrated entanglement of the emitted light and propelled its technological prospects. Manipulation of single or multiple photons has been achieved with more complex systems such as a TLS coupled to a cavity (*16*–*18*). For example, Faraon *et al.* (*19*) have reported photon statistics influenced by a strongly coupled cavity, interpreted as a “photon-induced tunneling.” Multiphoton correlations have been observed in the emission of a strongly driven TLS when its spectrum is frequency resolved (*20*). In the weak coupling regime, strong bunching in the second-order correlation arises from photon-number–dependent transmission and reflection (*21*, *22*), which results from internal interference in the system but limits the tunability. Photon statistics can be also controlled with an ensemble of atoms mediating interference (*23*, *24*) by taking advantage of many-body enhancement. However, the multiphoton dynamics of the simplest system in quantum optics remains largely unexplored, leaving gaps in the understanding of the underlying physics.

In this work, we investigate the fundamental quantum interference of multiphoton fluctuations with a classical mean field in the resonance fluorescence of a TLS under weak driving. We observe that antibunching in all multiphoton correlations (experimentally up to three-photon) turns into superbunching of all orders when the system is reduced to its quantum fluctuations. This result is achieved by disrupting the interferences with a full and independent control of the mean field. A precise admixture of the classical and quantum fields realizes individual suppression of photon numbers, revealing how the multiphoton coincidences behave independently from each other. This tunability opens up promising possibilities for harnessing multiphoton physics.

## RESULTS

### Classical coherent mean field and quantum fluctuations in resonance fluorescence

**Fig. 1.**
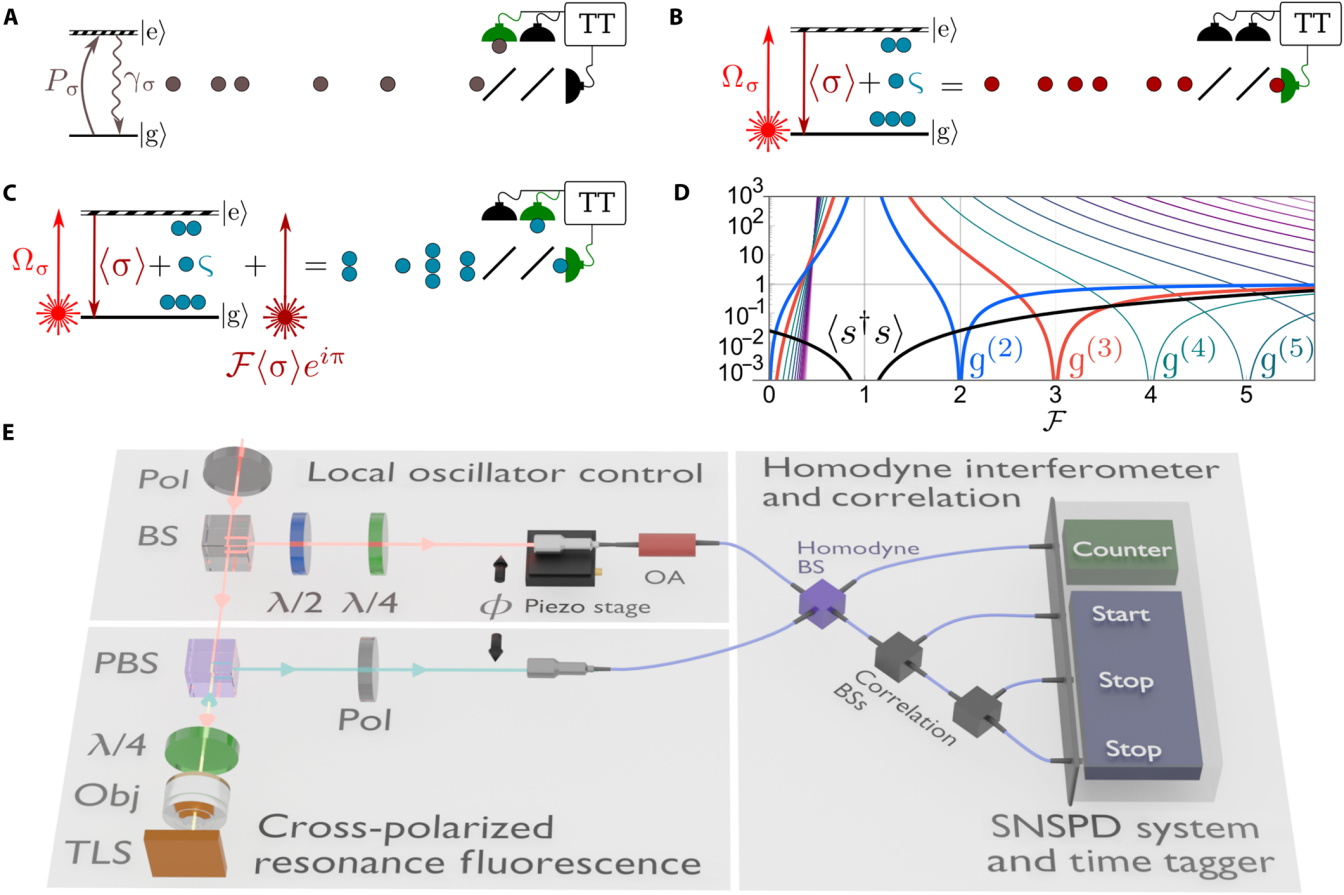
Multiphoton fluctuations in single-photon emission from a coherently driven TLS. (**A**) For incoherent driving $P_{\sigma }$, a TLS σ allows only one excitation at a time, leading to single-photon emission characterized by an extended Hanbury Brown–Twiss setup. TT, time-tagging unit. (**B**) Under coherent driving $\Omega_{\sigma }$, however, the single-photon emission arises from interferences between the coherent mean field 〈σ〉 and the quantum fluctuations ς. The probability amplitudes of multiphotons are canceled out by the destructive interferences with the mean field. (**C**) Disrupting the interferences by adding an external coherent field $F\langle \sigma \rangle e^{i\pi }$ can bring the multiphotons to light or suppress a given *n*-photon emission in an otherwise almost coherent field. (**D**) Glauber’s $g^{\left(n\right)}$ correlators provide suitable observables for the characterization of quantum light. In the Heitler regime, they all diverge at F=1 when the external field cancels 〈σ〉—in which case multiphoton emission to all orders is observed from the TLS, beside its emission $\langle s^{†}s\rangle$ approaching 0. At F=n, each $g^{\left(n\right)}$ is individually suppressed. (**E**) Experimental realization of the scheme by combining cross-polarized resonance fluorescence of a single quantum dot and a homodyne setup. An external coherent field—the local oscillator (LO)—is controlled in polarization, phase ϕ, and intensity $\propto F^{2}$. The homodyned signal is fed to a detection system, which allows us to study correlations with stabilized phase control. Pol, polarizer; BS, beam splitter; PBS, polarizing beam splitter; λ/2, half-waveplate; λ/4, quarter-waveplate; Obj, objective lens; OA, optical attenuator; SNSPD, superconducting nanowire single-photon detector.

The TLS, with its operator σ≡∣g〉〈e∣, is the most fundamental quantum emitter. Its only transition from the excited ∣e〉 to the ground ∣g〉 state can be saturated, resulting in a stream of antibunched photons. While this paradigmatic description for single-photon emission from a TLS is accurate in cases such as with incoherent excitation (Fig. 1A), under coherent driving, an entirely different scenario arises (Fig. 1B). The coherent driving of an oscillator leads to a coherent response, even when this oscillator is quantum (*25*), and small nonlinearities can be dealt with perturbatively in the form of fluctuations. The fluctuations are obtained by subtracting the mean field 〈σ〉 of the system from the TLS operator σ

ς≡σ−〈σ〉 (1)

where ς is the quantum fluctuation operator. When the driving $\Omega_{\sigma }$ is weak compared to the radiative decay rate $\gamma_{\sigma }$ of the emitter, the ratio between the intensity of the mean field ${|\;\langle \sigma \rangle \;|}^{2}$ and the intensity of the fluctuations $\langle \varsigma^{†}\varsigma \rangle$ can be made arbitrarily large

$$\langle \varsigma^{†}\varsigma \rangle \ll {|\;\langle \sigma \rangle \;|}^{2}$$ (2)

In this so-called Heitler regime (*1*), i.e., when Ω≪1, where $\Omega \equiv \Omega_{\sigma }/\gamma_{\sigma }$, the total intensity $\langle \sigma^{†}\sigma \rangle ={|\;\langle \sigma \rangle \;|}^{2}+\langle \varsigma^{†}\varsigma \rangle$ is composed of (*26*)

$$\begin{array}{c} {|\;\langle \sigma \rangle \;|}^{2}={\left(\frac{2\Omega }{1+8\Omega^{2}}\right)}^{2}\text{and}\;\langle \varsigma^{†}\varsigma \rangle =8\Omega^{2}{|\;\langle \sigma \rangle \;|}^{2} \end{array}$$ (3)

In a classical setting, when fluctuations are considerably smaller than the mean field, they are regarded as negligible or treated as perturbative corrections. In a fully quantum description, this perturbative picture breaks down regardless of the quantitative imbalance, and what occurs is a much more marked excitation of the quantum field into a superposition of all its possible multiphoton states. This outcome is surprising, as one might expect that in this weak driving regime, multiphotons would play no role whatsoever. As far as single-photon emission is concerned, interferences of the quantum fluctuations with the classical mean field result in the lastly observed emission of single photons, out of a quantum field of multiphotons whose probability amplitudes are canceled by the interferences. These multiphotons can be revealed by disrupting their interferences. Experimentally, this can be achieved by controlling the mean field—a coherent component—through the so-called homodyne technique (*27*). It consists in admixing a local oscillator (LO) with the signal to precisely adjust the coherent fraction. This technique proved extremely effective to observe small quantum effects obscured by a strong classical field (*28*–*30*). Here, one can seize control of the coherent field to unknit the single-photon emission into multiphotons (Fig. 1C). The external LO field is represented as a coherent state

$$|F\langle \sigma \rangle e^{\mathrm{i\phi}}\rangle$$ (4)

whose amplitude we write as a factor F of 〈σ〉. The relative phase ϕ=π is set to be opposite to the phase of the mean field. As a result, the intensity of the total signal $s\equiv \sigma +F\langle \sigma \rangle e^{i\pi }$, which is, in general,

$$\langle s^{†}s\rangle ={\left(F-1\right)}^{2}{|\;\langle \sigma \rangle \;|}^{2}+\langle \varsigma^{†}\varsigma \rangle$$ (5)

reduces to the quantum fluctuations $\langle \varsigma^{†}\varsigma \rangle$ when F=1.

### Multiphoton observables of homodyned signal

Next, we show how the small fluctuations govern the multiphoton physics of the system. The best way to characterize quantum light is through the standard observables in quantum optics: Glauber’s *n*th-order correlation functions $g^{\left(n\right)}\left(\tau_{1},\ldots ,\tau_{n-1}\right)$. They quantify the density of *n*-photon detections separated by times $\tau_{i}$. The joint detection of *n* photons, i.e., with $\tau_{i}=0$ for all *i*, measures by how much a coincidence is magnified (or suppressed if <1) as compared to an uncorrelated signal of same intensity. For the problem at hand of unleashing multiphoton emission from a TLS by admixing an external LO field, the *n*-photon coincidences can be obtained exactly for any driving. The dynamics of the multiphoton observables exhibit a stronger transition between antibunching and bunching for higher photon orders as the driving strength decreases (see Materials and Methods for details). In the Heitler limit of Ω→0, they read

$$g^{\left(n\right)}\left(0\right)=\frac{F^{2\left(n-1\right)}{\left(F-n\right)}^{2}}{{\left(F-1\right)}^{2n}}$$ (6)

From the denominator, one can see that these multiphoton observables, shown in Fig. 1D, diverge for all *n* when F=1. They are superbunched for all photon numbers according to Eq. 6. This so-called unconventional bunching (*31*) occurs when the mean field 〈σ〉 is canceled completely from the signal, leaving only the quantum fluctuations ς. Subtracting the mean field from a TLS leads to strong multiphoton emission to all orders.

Now turning to the numerator of Eq. 6, one can see that the multiphoton correlations have two zeros: the first one for all *n* at F=0, i.e., without an external field. This case is the standard resonance fluorescence, which exhibits both the conventional antibunching (*31*)—single-photon emission—and subnatural linewidth (*32*, *33*), although these two properties cannot be observed simultaneously (*34*–*37*).

More notable features occur with the second zero in Eq. 6, which is *n* dependent with $g^{\left(n\right)}\left(0\right)\to 0$ at F=n. Although F is a continuous variable, as befits a classical field, it triggers a strong response of the system when taking integer values, which is a manifestation of the interplay between interfering continuous and quantized fields. Unlike the previous case with F=0, these multiphoton resonances are not degenerate. One can suppress any given photon number individually without strongly affecting the other correlators, realizing the unconventional antibunching (*31*). In particular, one can suppress two-photon emission only, i.e., $g^{\left(2\right)}\left(0\right)\to 0$ at F=2. In this case, because of the proximity to the divergence at F=1, all the other photon-number coincidences remain much larger than would be expected on accounts of random events alone, with $g^{\left(n\right)}\left(0\right)\gg 1$ for n≥3. In other words, the suppression of two-photon coincidences does not preclude increased coincidences of higher numbers of photons such that

$$g^{\left(2\right)}\left(0\right)\ll g^{\left(3\right)}\left(0\right)$$ (7)

At F=3, one can realize a suppression of the three-photon coincidences without strong suppression of the two-photon ones, $g^{\left(3\right)}\left(0\right)\to 0$ while $g^{\left(2\right)}\left(0\right)⪅1$, and reverse the trend of Eq. 7, i.e., qualitatively

$$g^{\left(2\right)}\left(0\right)\gg g^{\left(3\right)}\left(0\right)$$ (8)

### Experimental realization of multiphotons from single-photon emission

**Fig. 2.**
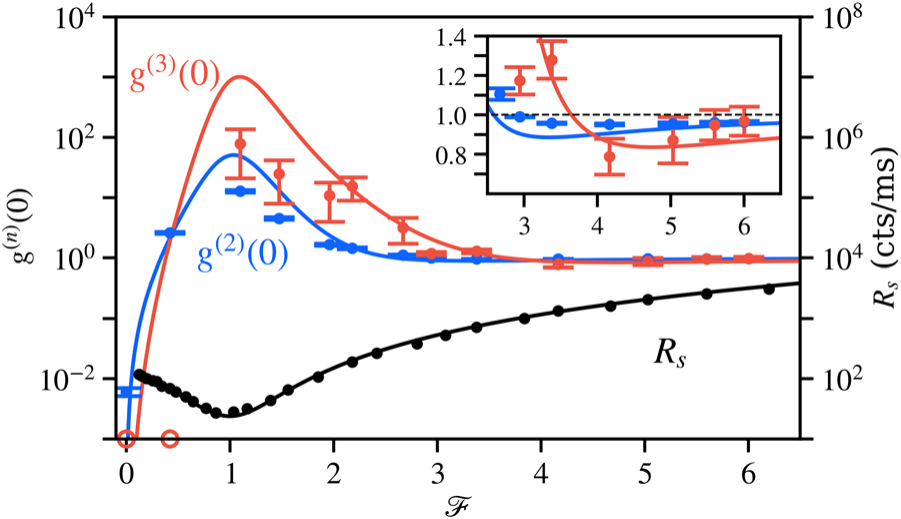
Controlling and revealing multiphoton dynamics in a single-photon source. Experimental results (symbols) and theoretical predictions (solid lines) for count rate (black), along with $g^{\left(2\right)}\left(0\right)$ (blue) and $g^{\left(3\right)}\left(0\right)$ (red) of the homodyned signal, as functions of the LO field amplitude F under a driving Ω≈0.15. At F=0, without admixing with an external field, multiphoton events remain virtual due to destructive interferences of their probability amplitudes, resulting in the emission of single photons. At F=1, the external field cancels the mean field of the system and thus lays bare its quantum fluctuations. The multiphoton nature of the fluctuations is revealed in strong $g^{\left(2\right)}\left(0\right)$ and $g^{\left(3\right)}\left(0\right)$ superbunching despite a drop of the count rate by about an order of magnitude. For a sizable coherent field, one can suppress *n*-photon emission independently. The cases n=2 and n=3 are shown and magnified in the inset, confirming the transition from two-photon suppression to three-photon suppression. The two undetermined cases with $g^{\left(3\right)}\left(0\right)=0$, arising from zero coincidence events throughout the entire integration time, are shown on the horizontal axis.

In our experiment, the TLS is realized with a single InGaAs quantum dot under weak driving (see Materials and Methods for details). Our setup with multiphoton coincidence counting units to characterize the homodyned signal is sketched in Fig. 1E. In Fig. 2, we study the count rate and multiphoton coincidences, as the mean field is manipulated. Experimentally, the measured count rate $R_{s}$ of the admixture is proportional to the theoretical quantity $\langle s^{†}s\rangle$, scaled by overall experimental efficiency, emitter decay rate, and other relevant factors. The count rate as a function of the external field F (black symbols) allows us to access the count rate of the coherent field, $R_{\langle \sigma \rangle }\propto \gamma_{\sigma }{|\;\langle \sigma \rangle \;|}^{2}$, and that of the quantum field, $R_{\varsigma }\propto \gamma_{\sigma }\langle \varsigma^{†}\varsigma \rangle$, as well as to extract the applied driving strength experienced by the system. We obtain $R_{\langle \sigma \rangle }\approx 252.2$ counts (cts)/ms and $R_{\varsigma }\approx 47.5$ cts/ms at the driving Ω≈0.15 (Supplementary Text). For the given driving, the contribution of the coherent mean field to the total emission is about 84%, satisfying Eq. 2. As F increases from 0 to 1, the count rate of the admixture decreases by roughly an order of magnitude, as the coherent emission is gradually removed. When only the fluctuations remain in the signal, multiphotons emerge with strong bunching correlations. Theoretically, we observe $g^{\left(n+1\right)}\left(0\right)\gg g^{\left(n\right)}\left(0\right)\gg 1$ for all n≥2, and experimentally, we confirm $g^{\left(3\right)}\left(0\right)\gg g^{\left(2\right)}\left(0\right)\gg 1$ at F=1 in Fig. 2. While this quantum effect becomes more pronounced as the driving decreases, in the laboratory, the Heitler limit is an asymptotic ideal, which must be compounded with experimental limitations such as efficiency and stability. For finite driving, we observe that the correlations retain the same structure but become smoother, decrease in contrast, and shift in position.

The nondegenerate multiphoton antibunching resonances are observed with up to three photons in the inset of Fig. 2. These resonances at the finite driving are not as prominent as in the mathematical limit. However, all the qualitative relationships of Eqs. 7 and 8 are satisfied and in good agreement with the theoretical prediction for this driving. We observe two-photon antibunching and three-photon bunching at F=3.38. Namely, with $g^{\left(2\right)}\left(0\right)=0.957\pm 0.004$ and $g^{\left(3\right)}\left(0\right)=1.28\pm 0.10$, we have $g^{\left(2\right)}\left(0\right)<1<g^{\left(3\right)}\left(0\right)$. At F=4.17, we confirm the opposite stronger three-photon suppression with $g^{\left(2\right)}\left(0\right)=0.951\pm 0.004$ and $g^{\left(3\right)}\left(0\right)=0.79\pm 0.09$. Consequently, we demonstrated that multiphoton coincidences can be enhanced or suppressed, together or independently, depending on how the mean field ties them together.

## DISCUSSION

### Nonclassical photon statistics from quantum fluctuations

**Fig. 3.**
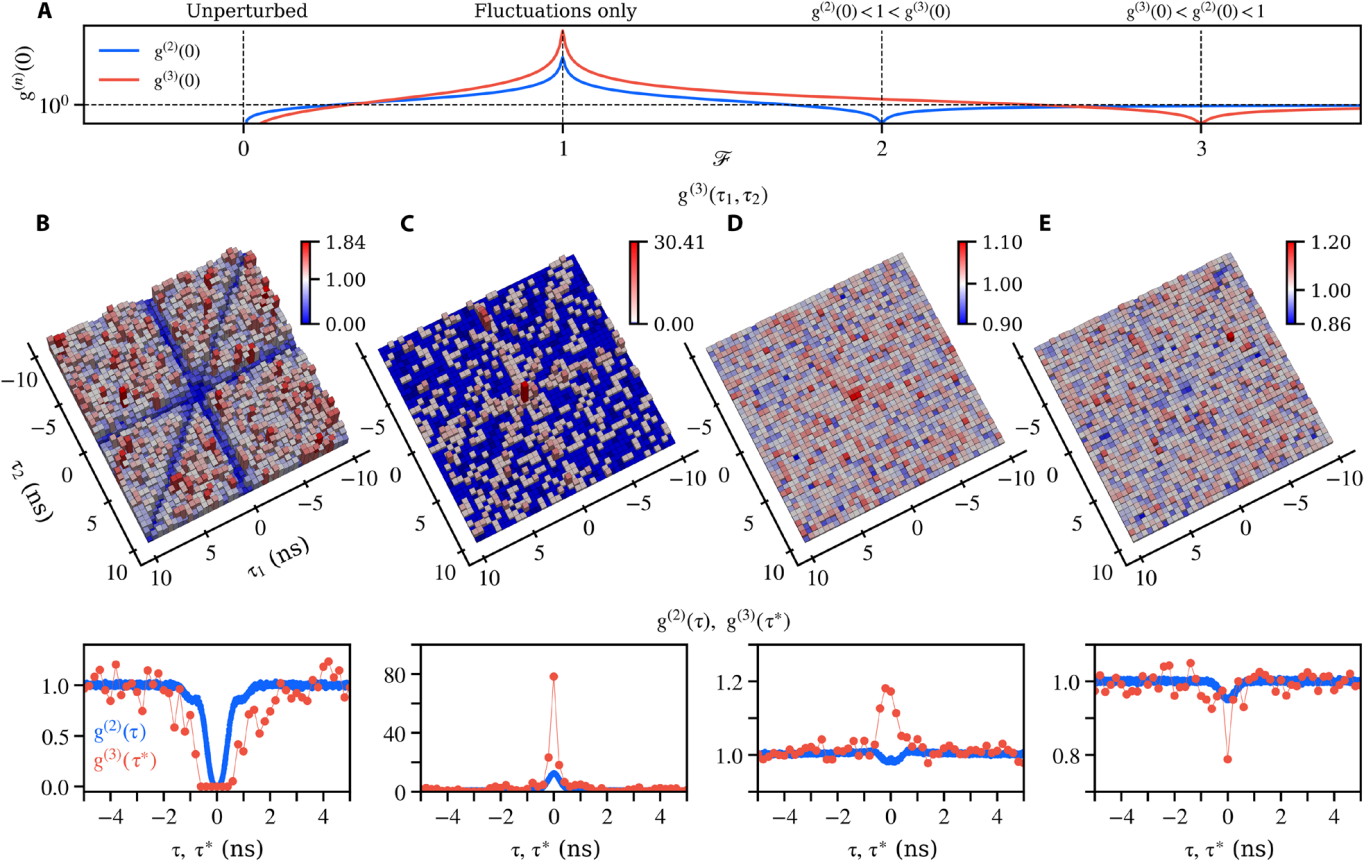
Measured second- and third-order correlations of the signal under homodyne mean-field control. (**A**) Calculated multiphoton observables in the Heitler limit Ω→0. (**B** to **E**) For Ω≈0.15 normalized third-order coincidences $g^{\left(3\right)}\left(\tau_{1},\tau_{2}\right)$ with time delays $\tau_{1}$ and $\tau_{2}$ (second row) and their integrated results $g^{\left(3\right)}\left(\tau^{*}\right)$ with second-order correlation $g^{\left(2\right)}\left(\tau \right)$ (third row) for four configurations of interest. (B) With no external field, F=0, all multiphoton probabilities are suppressed, producing single-photon emission. In $g^{\left(3\right)}\left(\tau_{1},\tau_{2}\right)$, this is visible as three depleted lines [$g^{\left(2\right)}\left(0\right)\ll 1$] and a wide dip at their intersection [$g^{\left(3\right)}\left(0,0\right)\ll 1$] in an otherwise uncorrelated background, satisfying $g^{\left(3\right)}\left(0\right)\ll g^{\left(2\right)}\left(0\right)\ll 1$. (C) Canceling the mean field, with F=1, we observe strong multiphoton bunching from the quantum fluctuations, visible as three lines of the signal standing on the vacuum. This realizes $g^{\left(3\right)}\left(0\right)\gg g^{\left(2\right)}\left(0\right)\gg 1$. (D) Changing the sign of the mean field realizes the counterintuitive relation $g^{\left(2\right)}\left(0\right)<1<g^{\left(3\right)}\left(0\right)$. Two-photon emission is suppressed, but not higher-order emissions, thus disqualifying this regime as a single-photon source. (E) Individual suppression of the three-photon component. Further increasing the mean field realizes the next case where $g^{\left(3\right)}\left(0\right)<1$ while $g^{\left(2\right)}\left(0\right)⪅1$.

To discuss the multiphoton physics underlying these counterintuitive quantum phenomena in more detail, we examine four distinct regimes with a theoretical overview (Fig. 3A) across continuous variations of F in the Heitler limit. Experimental results of $g^{\left(3\right)}\left(\tau_{1},\tau_{2}\right)$, where $\tau_{1}$ and $\tau_{2}$ are the time differences of events detected by the second and third detectors relative to the first, are shown in Fig. 3 (B to E) under a finite driving Ω≈0.15, along with $g^{\left(2\right)}\left(\tau \right)$ and radially integrated $g^{\left(3\right)}\left(\tau^{*}\right)$, where $\tau^{*}$ represents the effective time delay for three-photon events as $|\;\tau^{*}\;|=\sqrt{\tau_{1}^{2}+\tau_{2}^{2}}$ (see Materials and Methods for details).

Our observations are unequivocal: The leftmost column describes the usual Heitler regime of resonance fluorescence. At F=0, while the emission is predominantly coherent, it is antibunched in all multiphoton correlators. The antibunching in the second order, $g^{\left(2\right)}\left(0\right)=0.006\pm 0.001$, is also seen in $g^{\left(3\right)}\left(\tau_{1},\tau_{2}\right)$ of Fig. 3B as three lines with vanishing coincidences across the whole landscape when two of the three detectors are triggered at the same time, i.e., $\tau_{1}=0$, $\tau_{2}=0$ or $\tau_{1}=\tau_{2}$. At their intersection, $\tau_{1}=\tau_{2}=0$, no photon triplet within a 1.2-ns time delay [corresponding to the center plateau of the integrated ]$g^{\left(3\right)}\left(\tau^{*}\right)$ was observed over the entire integration time (>19 hours). For an uncorrelated signal of the same intensity, approximately 12 three-photon coincidence events would be expected from the averaged uncorrelated three-photon events ${\bar{G}}^{\left(3\right)}\left(\infty \right)=2.02$ for 200-ps binning.

The second column, Fig. 3C, describes the case at F≈1, showcasing our homodyne technique that gives us access to the quantum fluctuations ς hidden in the undisrupted case. How the quantum signal looks like in $g^{\left(3\right)}\left(\tau_{1},\tau_{2}\right)$ landscape is notable: The considerable drop in classical emission but the persistence of simultaneous two- and three-photon emission produces bunched diagonals surrounded by the absence of coincidences from the vacuum field. Although the three-photon coincidence $g^{\left(3\right)}\left(0,0\right)$, as a third-order process, is measured from a much more scarce signal than $g^{\left(2\right)}\left(0\right)$, its much stronger deviation from the classical case makes its features more discernible. We obtain $g^{\left(2\right)}\left(0\right)=12.7\pm 0.6$ and $g^{\left(3\right)}\left(0\right)=78\pm 57$, confirming the multiphoton nature of the fluctuations. Our findings can be seen directly from the photon number probability distribution, which can be extracted from the Glauber correlators (*38*). The probability of *n*-photon coincidences p(n) of the homodyned signal is given by the Poissonian distribution of the external LO modulated by the quantum fluctuations (see Materials and Methods for details). At F=1, the TLS could be seen as a single-photon filter of the external LO, suppressing the single-photon emission in the admixture, while the probability of two-photon coincidences p(2) is identical to that of the LO as discussed earlier (*21*, *23*, *39*). However, this filter picture does not hold for higher photon numbers. Instead, the weak signal displays ${\left(n-1\right)}^{2}$ times more *n*-photon coincidences than the external field does. This shows that, from three photons upward, a coherent state can extract more multiphoton events from the quantum fluctuations than it has itself. Thus, there exists a multiphoton amplification for n≥3. The one-photon probability still remains dominant among all *n*-photon probabilities even at its strongest suppression at F=1 .

The third and fourth columns, where the mean field is again sizable, compellingly show the qualitative (but not yet quantitative) relationships 7 and 8, which complete the versatility of the interplay between the mean field and the fluctuations. Bunching and antibunching of photon triplets are directly visible at the center of the three-photon landscapes $g^{\left(3\right)}\left(\tau_{1},\tau_{2}\right)$ in Fig. 3 (D and E), respectively. By introducing more external field as the LO amplitude F increases from 2.94 to 4.17, the antibunching resonance $g^{\left(n\right)}\left(0\right)<1$ shifts from n=2 to n=3 as shown by the transition of $g^{\left(3\right)}\left(0\right)$ from 1.17±0.07 to 0.79±0.09, while $g^{\left(2\right)}\left(0\right)$ remains below 1. In Fig. 3D, one can see this independent behavior of the multiphoton observables that suppresses two-photon events, while three-photon ones become more likely than an uncorrelated signal with same intensity. With further increase of the external field, three-photon events are the most strongly suppressed among all *n*. This is counterintuitive because one would expect the impact of the fluctuations to become negligible due to a strong external field at F≫1. However, instead of smothering the quantum attributes by making the fluctuations even more negligible, the classical field singles out strong $g^{\left(n\right)}\left(0\right)\ll 1$ for high-order n≥2 quantum resonances, with no theoretical upper limit either on *n* or on the total field intensity. In this regime, a strong destructive quantum interferences occur selectively for a specific photon number that is determined by the amplitude of the external field.

### Multiphoton observables as a function of driving

**Fig. 4.**
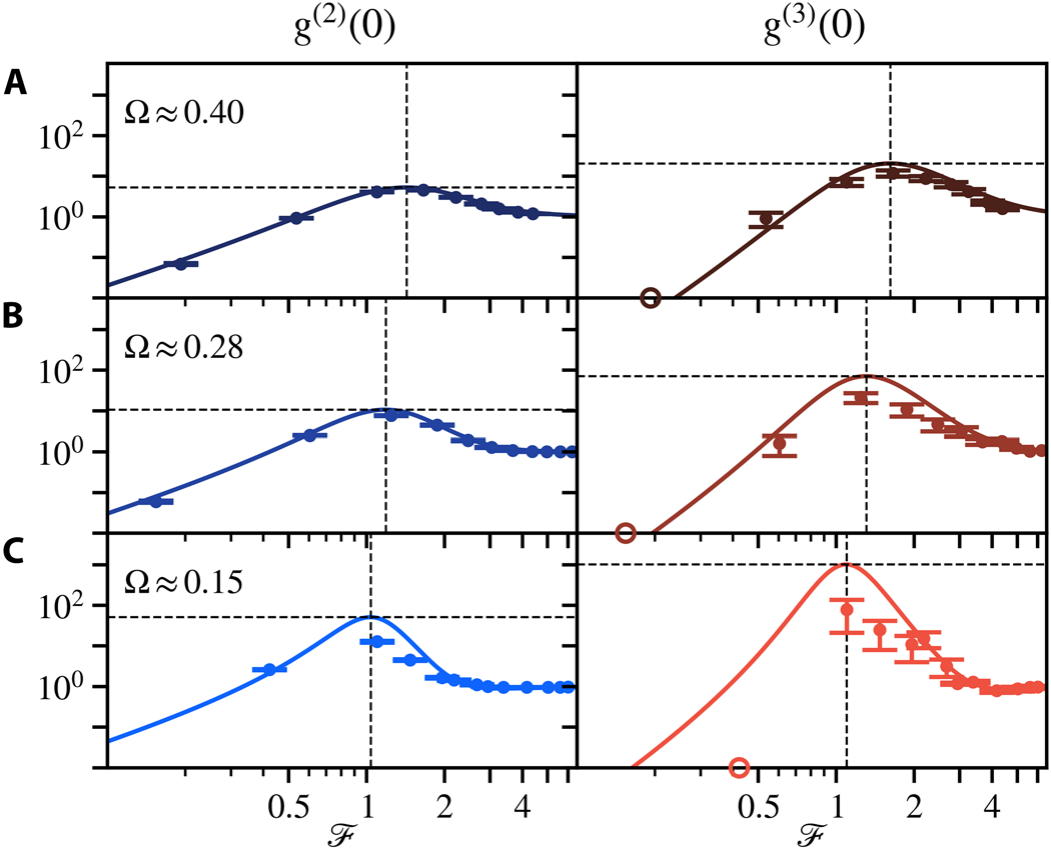
Enhanced multiphoton dynamics near the Heitler limit. Power-dependent correlations of $g^{\left(2\right)}\left(0\right)$ (left) and $g^{\left(3\right)}\left(0\right)$ (right) with decreasing driving of (**A**) Ω≈0.40, (**B**) Ω≈0.28, and (**C**) Ω≈0.15. The multiphoton correlations of the fluctuations at F≈1 increases as driving decreases, i.e., as the system approaches the Heitler limit, also with resonances converging to each other and toward F=1. Theoretical and experimental results are given as solid lines and symbols, respectively, and dashed guidelines represent the strongest bunching resonances. All the data presented in the main text are for the lowest driving of (C) where the correlations exhibit the strongest variation. As a result of setup instability over the long acquisition time required because of the weak signal, deviations from the theory are also the largest for this driving strength. The undetermined cases with $g^{\left(3\right)}\left(0\right)=0$, arising from zero coincidence events throughout the entire integration time, are shown on the horizontal axis.

In Fig. 4, we provide evidence for such nonclassical observations being enhanced in the deeper Heitler regime. The correlations at three driving strengths (Ω≈0.40,0.28,and0.15) show that the multiphoton effects are indeed magnified as the system approaches the lower driving case, although the intensity contribution of the quantum fluctuations decreases ($\langle \varsigma^{†}\varsigma \rangle /\langle \sigma^{†}\sigma \rangle$ of 56, 38, and 16%; Supplementary Text). Furthermore, the two- and three-photon resonances converge toward the common theoretical value F=1. The largest deviation between the measured values and the theoretical model is found with the smallest driving, which we primarily attribute to the limited setup stability during the long integration time of 277 hours for Ω≈0.15 (Supplementary Text). The larger signal allows us to conduct the measurements over smaller periods of time (25.2 hours for Ω≈0.28 and 4.5 hours for Ω≈0.40), thereby mitigating this limitation. Consequently, there is a closer agreement with the theoretical model at higher powers, where quantum effects are, however, attenuated. One could, with brighter emitters and more efficient and stable setups, optimize the features we have reported by further reducing Ω, i.e., accessing deeper the Heitler regime.

In conclusion, we experimentally demonstrated the external control of the quantum emission from a TLS under weak coherent driving by observing photon correlations up to the third order. This control unveils the intrinsic multiphoton nature of the paradigmatic, fundamental, and simplest quantum optical emitter, producing both antibunching and bunching across all orders and their independent suppression. The multiphoton physics of the system is governed by quantum interferences of multiphoton fluctuations with a coherent mean field, which we manipulate independently. These quantum effects become increasingly pronounced, as the system approaches the deeper Heitler regime. This multiphoton aspect similarly applies to quantum emitters in cavities, coupled TLSs, and strongly correlated phases in condensed matter systems. Our results open the door to further exploit such effects as a resource, for example, feeding a deterministic photon sorting system (*40*) for photonic phase transistors that take advantage of the multiphoton amplification that we have identified for n≥3 or through the realization of a simultaneous subnatural linewidth single-photon source (*34*). Building on our demonstration, we anticipate that mean-field control will serve as an universal key for unlocking multiphoton emission in coherently driven quantum systems and for enabling the generation of nonclassical light beyond single photons.

## MATERIALS AND METHODS

### Theoretical model for multiphoton observables

A TLS under coherent driving at resonance is modeled by the Hamiltonian, $H=\Omega_{\sigma }\left(\sigma^{†}+\sigma \right)$. Solving the master equation, $\partial_{t}\rho =i\left[\rho ,H\right]+\left(\gamma_{\sigma }/2\right)L_{\sigma }\rho$, in the Lindblad form, where the superoperator $L_{\sigma }\rho =2{\sigma \rho \sigma }^{†}-\sigma^{†}\sigma \rho -{\rho \sigma }^{†}\sigma$, yields the following solution for the steady state

$$\rho =\left(\begin{array}{cc} 1-\langle n_{\sigma }\rangle & {\langle \sigma \rangle }^{*}\\ \langle \sigma \rangle & \langle n_{\sigma }\rangle \end{array}\right)$$ (9)

where

$$\langle n_{\sigma }\rangle =\frac{4\Omega_{\sigma }^{2}}{\gamma_{\sigma }^{2}+8\Omega_{\sigma }^{2}}\;\text{and}\;\langle \sigma \rangle =\frac{2i\Omega_{\sigma }\gamma_{\sigma }}{\gamma_{\sigma }^{2}+8\Omega_{\sigma }^{2}}$$ (10)

are the population and the mean field of the system, respectively. The correlation functions of the homodyned signal can be derived by substituting σ with $s=\sigma +F\langle \sigma \rangle e^{\mathrm{i\phi}}$ (*41*)

$$\begin{array}{c} G^{\left(n\right)}\left(0\right)=\langle s^{†n}s^{n}\rangle =\\ {\left(F^{2}{|\langle \sigma \rangle |}^{2}\right)}^{n}{\left(F\gamma_{\sigma }\right)}^{-2}\left[\left(n^{2}+F^{2}\right)\gamma_{\sigma }^{2}+8n^{2}\Omega_{\sigma }^{2}+2nF\gamma_{\sigma }^{2}\text{cos}\left(\phi \right)\right] \end{array}$$ (11)

from which the Glauber’s correlation functions read

$$\begin{array}{c} g^{\left(n\right)}\left(0\right)=\frac{\langle s^{†n}s^{n}\rangle }{{\langle s^{†}s\rangle }^{n}}\\ =\frac{{\left(F\gamma_{\sigma }\right)}^{2\left(n-1\right)}\left[\left(n^{2}+F^{2}\right)\gamma_{\sigma }^{2}+8n^{2}\Omega_{\sigma }^{2}+2nF\gamma_{\sigma }^{2}\text{cos}\left(\phi \right)\right]}{{\left[F^{2}\gamma_{\sigma }^{2}+2F\gamma_{\sigma }^{2}\text{cos}\left(\phi \right)+\left(\gamma_{\sigma }^{2}+8\Omega_{\sigma }^{2}\right)\right]}^{n}} \end{array}$$ (12)

This result simplifies to Eq. 6 for vanishing driving ($\Omega_{\sigma }/\gamma_{\sigma }\to 0$) and out-of-phase (ϕ=π) condition.

The photon number probability distribution p(n) of the signal, representing the diagonal elements of the effective quantum state, accounts for the correlations of the admixture and can be reconstructed from the Glauber’s correlators as (*38*)

$$p\left(n\right)=\sum_{k=0}^{\infty }\frac{{\left(-1\right)}^{k}}{k!}\frac{G^{\left(n+k\right)}\left(0\right)}{n!}$$ (13)

Substituting $G^{\left(n\right)}\left(0\right)$ in this formula by Eq. 11, the analytical solution of the photon number distribution can be obtained regardless of the driving strength. However, for simplicity, we consider the limiting case of weak driving

$$p\left(n\right)=p_{\text{coh}}\left(n\right)M_{F}\left(n\right)$$ (14)

where $p_{\text{coh}}\left(n\right)=\langle n|F\langle \sigma \rangle e^{\mathrm{i\phi}}\rangle$ is the Poisson distribution of the external coherent state as shown in Eq. 4. $M_{F}\left(n\right)$ is a multiphoton quantum correction that modulates this coherent state

$$\begin{array}{c} M_{F}\left(n\right)\equiv {\left(1-\frac{n}{F}\right)}^{2}-\left(1+2n-2F-\frac{2n^{2}}{F^{2}}\right){\left(2\Omega \right)}^{2}+o\left(\Omega^{4}\right) \end{array}$$ (15)

This description of the quantum interference predisposes one to see the TLS as a photon-number–sensitive filter; however, as discussed in the text, this is, in fact, more similar to a multiphoton amplifier (from three-photon upward) because more multiphotons can be emitted than are available anywhere in the system .

### Quantum dot as a TLS

The experiments are performed with a single self-assembled quantum dot, where a neutral exciton exhibits a fine-structure splitting of 790 MHz. One dipole of the neutral exciton transitions, with an emission wavelength of 910 nm, an almost-transform limited linewidth of 770 MHz in absorption spectrum and a radiative lifetime of 216 ps, is driven by a continuous-wave laser. The narrow laser’s linewidth (<10 kHz) allows for resonant driving of the single dipole in our quantum dot, thereby realizing a TLS. The sample is cooled down to 4.2 K in a helium dip stick. A Schottky diode structure embedding the studied quantum dot allows us to stabilize the electronic environment and to fine-tune the emission wavelength of the system via the quantum-confined Stark effect (*42*). Below the quantum dot layer, a distributed Bragg reflector enhances the collection of resonance fluorescence from the system. The reflection of the excitation laser is effectively suppressed by cross-polarized excitation and detection (*43*).

### Homodyne mean-field engineering

As shown in Fig. 1E, our homodyne interferometer is constructed similarly to (*29*); however, instead of using the reflection of the excitation as a LO input, we pick off the excitation beam before interaction with the sample. This ensures that the LO remains a coherent state, free from any emission by the system. The polarization of the LO is carefully controlled by a set of motorized half- and quarter-waveplates to align with the polarization of the collected system emission. We control the intensity of the LO, which is proportional to $F^{2}$, using a fiber-based optical attenuator. The phase difference ϕ between the emission and the LO is either scanned or stabilized upon measurement requirements by adjusting the propagation path length of the LO with a linear translation stage. The stabilization during correlation measurements is managed by a software Proportional-Integral-Derivative control. In each control cycle (10 ms), interference visibility is calculated from the detected counts of all channels, and the stage position is adjusted to maintain a given target visibility. For passive stabilization, we place our setup in a homemade stabilization box whose interior is covered with acoustic absorption foam to reduce the impact of environmental vibrations. By interfering the LO and the system emission in a polarization-maintaining fiber-based beam splitter, we minimize possible degradation of signal such as polarization rotation and mode mismatch caused from environmental fluctuations, e.g., temperature drift and mechanical vibrations.

### Three-photon correlation

To examine the second- and third-order correlations, one output of the homodyne beam splitter in Fig. 1E, where the signal of interest is desired, is split by two cascaded 50:50 fiber-based beam splitters and collected by three superconducting nanowire single-photon detectors, implementing an extended Hanbury Brown–Twiss setup. Time tags of detection events are recorded by a multichannel time tagger with an overall timing jitter of ~20 ps. This configuration allows for the measurement of the unnormalized three-photon correlation $G^{\left(3\right)}\left(\tau_{1},\tau_{2}\right)$ through a two-dimensional histogram. These time-correlated multiphoton measurements are technically challenging because we operate with a single TLS, which we furthermore push in a regime of vanishing classical emission. A large bin size in these correlation measurements can mitigate limited signal counts, but it also reduces the timing resolution. As a result, features such as bunching or antibunching become less noticeable, similarly to the effects of detector timing jitter (*44*). A bin size of 500 ps is chosen for the $g^{\left(3\right)}\left(\tau_{1},\tau_{2}\right)$ results presented in Fig. 2, achieving visible features with a reasonable signal-to-noise ratio. We maximize the visibility of the signal and further reduce the bin size by performing a radial integration along the antidiagonal line ($\tau_{1}=-\tau_{2}$) in $G^{\left(3\right)}\left(\tau_{1},\tau_{2}\right)$ and obtain $g^{\left(3\right)}\left(\tau^{*}\right)$ by normalizing the integrated $G^{\left(3\right)}\left(\tau^{*}\right)$ with the averaged uncorrelated events (Supplementary Text). As a result, the final values are extracted with a bin size of 200 ps for $g^{\left(3\right)}\left(\tau^{*}\right)$.
