# Supplementary material for: Unlocking multiphoton emission from a single-photon source through mean-field engineering
Source: Sci Adv. 2025 Oct 29;11(44):eadw3395. doi: 10.1126/sciadv.adw3395 (PMC13141893; doi:10.1126/sciadv.adw3395)
Supplement: Supplementary file 1 — Supplementary Text Figs. S1 and S2 Tables S1 to S3 [file sciadv.adw3395_sm.pdf]

Science Advances
AAAS

Supplementary Materials for

Unlocking multiphoton emission from a single-photon source through mean-field engineering

Sang Kyu Kim *et al.*

Corresponding author: Sang Kyu Kim, sangkyu.kim@tum.de; Kai Müller, kai.mueller@tum.de;
Elena del Valle, elena.delvalle.reboul@gmail.com

Sci. Adv. **11**, eadw3395 (2025)
DOI: 10.1126/sciadv.adw3395

This PDF file includes:

Supplementary Text
Figs. S1 and S2
Tables S1 to S3

Supplementary Text

System characterization: mean field, fluctuations and driving

In our experimental configuration depicted in Fig. 1E in the manuscript, the system σ—the resonance fluorescence of our TLS—interferes with an external coherent field given by Eq. 4 in the homodyne beam splitter (BS). The two outputs of the BS are written as $s_+ = \left(\sigma + \mathscr{F}\langle\sigma\rangle e^{i\phi}\right)/\sqrt{2}$ and $s_- = \left(\sigma - \mathscr{F}\langle\sigma\rangle e^{i\phi}\right)/\sqrt{2}$ with variable phase ϕ. In the manuscript, the factor of $1/\sqrt{2}$ is omitted for simplicity. Their intensities are expressed with the TLS operator and the fluctuation operator as $\langle s_\pm{}^\dagger s_\pm\rangle = \frac{1}{2}\left[\langle\varsigma^\dagger\varsigma\rangle + |\langle\sigma\rangle|^2\left(1 \pm 2\mathscr{F}\cos(\phi) + \mathscr{F}^2\right)\right]$, with an interference term varying with phase and the LO amplitude $\mathscr{F}$. As mentioned in the manuscript, the measured count rates are proportional to the corresponding quantities. We can express the measured count rates, $R_{s\pm} \propto \gamma_\sigma\langle s_\pm{}^\dagger s_\pm\rangle$, of the two BS outputs as

$$R_{s\pm} = \frac{1}{2}\left[R_\varsigma + R_{\langle\sigma\rangle}\left(1 \pm 2\mathscr{F}\cos(\phi) + \mathscr{F}^2\right)\right]. \tag{S1}$$

One can extract the count rate of the coherent field, $R_{\langle\sigma\rangle} \propto \gamma_\sigma|\langle\sigma\rangle|^2$, and that of the fluctuations, $R_\varsigma \propto \gamma_\sigma\langle\varsigma^\dagger\varsigma\rangle$, by examining the $\mathscr{F}$- and ϕ-dependent admixture signal intensities given by Eq. S1. Experimentally, we drive a piezo stage to continuously scan the phase, by varying the path length difference between the LO and the emission of the system, while measuring count rates of the two outputs for a given driving Ω and a constant LO intensity $\propto \mathscr{F}^2$. An exemplary time-traced counter measurement result of the two outputs for a excitation power of 2 µW $\propto \Omega^2$ and a LO count rate of 189.1 cts/ms is shown in Fig. S1A. In Eq. S1, the maxima and minima occur with $\phi = n\pi$, where n is an integer value. The phase scan with a large range allows us to observe multiple extrema in the results. We evaluate the average maximum and minimum for the given parameters of $\mathscr{F}$ and the excitation power, by considering extrema in every oscillation period from the whole time-traced measurement. Next, we study the $\mathscr{F}$-dependency of these averaged maximum and minimum counts for the same driving and present the result in Fig. S1B. The minima and maxima are given by red and blue symbols, corresponding to the positive and negative sign in Eq. S1 with $\phi = \pi$. The equation is used as a fitting function with two fitting parameters R_ς and $R_{\langle\sigma\rangle}$. As a result, we obtain the count rate of the coherent mean field of the system $R_{\langle\sigma\rangle} \approx 252.2$ cts/ms and that of the fluctuations $R_\varsigma \approx 47.5$ cts/ms. An excellent agreement between measured data and solid fitting curves is found in the figure. The driving $\Omega = \sqrt{(\langle\varsigma^\dagger\varsigma\rangle)/(8|\langle\sigma\rangle|^2)}$ can be derived from

Eq. 3 in the main manuscript. Given the count rates of the two components, a driving $\Omega \approx 0.15$ is estimated. Similarly, we characterize the system with two different drivings used in the power series of Fig. 4. As a result, $R_{\langle\sigma\rangle} \approx 849.7\,\text{cts/ms}$ and $R_{\varsigma} \approx 521.4\,\text{cts/ms}$ for $\Omega \approx 0.28$ as well as $R_{\langle\sigma\rangle} \approx 1193.1\,\text{cts/ms}$ and $R_{\varsigma} \approx 1504.0\,\text{cts/ms}$ for $\Omega \approx 0.40$ are extracted.

Analysis of multiphoton correlations

To study the three-photon correlation, we extend the standard Hanbury Brown-Twiss setup with two outputs to the modified version with three outputs as shown in Fig. 1E. As mentioned in the manuscript, we characterize $G^{(3)}(\tau_1, \tau_2)$ based on the time difference between photon arrival times at the three detectors. To extract $g^{(3)}(\tau^*)$ from the time correlation measurement $G^{(3)}(\tau_1, \tau_2)$, we perform an analysis which projects the two-dimensional data (τ_1, τ_2-dependent) onto the one-dimensional space (τ^*-dependent). First, the obtained data are transformed from the Cartesian coordinate system with (τ_1, τ_2) to a polar coordinate system with (τ^*, θ). As an example, the $G^{(3)}(\tau_1, \tau_2)$ result for a driving $\Omega \approx 0.15$ without the external LO and its transformed data $G^{(3)}(\tau^*, \theta)$ are shown in Fig. S2A and B, respectively. For the transformation, we define $\tau^* = \tau_1/\cos\theta$, where θ is the angle with respect to the τ_1 axis in the Cartesian coordinate system. The transformation allows us to integrate the data along the θ axis, preserving the time parameter τ^* which effectively contains time delay information of both τ_1 and τ_2. Consequently, we can evaluate $G^{(3)}(\tau^*)$, where τ^* can be interpreted as the effective time delay of three subsequent detection events by all three detectors. Specifically, we define it as

$$G^{(3)}(\tau^*) = \int_{\vartheta}^{\pi/2-\vartheta} G^{(3)}(\tau^*, \theta)\, d\theta\,, \tag{S2}$$

where the integration range $(\vartheta, \pi/2 - \vartheta)$ is centered at $\pi/4$. The integration center angle $\pi/4$ corresponds to the antidiagonal line ($\tau_1 = -\tau_2$) in the Cartisean coordinate system, where the most uncorrelated events occur due to the largest time difference between τ_1 and τ_2 for a given τ^*. We carefully choose $\vartheta = \pi/12$, making the integration range wide enough to benefit from a large integration window, improvement of signal-to-noise ratio, while still narrow enough to exclude the three diagonals from the integration, namely, $G^{(3)}(0, \tau_2)$, $G^{(3)}(\tau_1, 0)$ and $G^{(3)}(\tau_1, \tau_1)$ lines representing two-photon coincidences. The normalized correlation is given by $g^{(3)}(\tau^*) = G^{(3)}(\tau^*)/\bar{G}^{(3)}_{\infty}$, where $\bar{G}^{(3)}_{\infty}$ is the averaged uncorrelated events at $|\tau^*| \gg 0$ which are positioned at both ends

of $G^{(3)}(\tau^*)$.

For the second-order correlation $g^{(2)}(\tau)$, we first extract the unnormalized $G^{(2)}(\tau)$ by analyzing the same raw time-tag data which are used for the $g^{(3)}(\tau^*)$ analysis. To improve and balance counts, we combine the two channels out of the three of the extended Hanbury Brown-Twiss setup which are passing two BSs in Fig. 1E. A cross correlation analysis is performed on the combined channel with the other to obtain $G^{(2)}(\tau)$. Similar to the normalization step for $g^{(3)}(\tau^*)$, we evaluate $g^{(2)}(\tau)$ by dividing $G^{(2)}(\tau)$ by $\bar{G}^{(2)}_\infty$. The standard deviation in the averaged uncorrelated events $\bar{G}^{(n)}_\infty$ and the Poissonian statistics of $G^{(n)}(0)$ are taken into account to calculate errors of $g^{(n)}(0)$ for $n = 2, 3$.

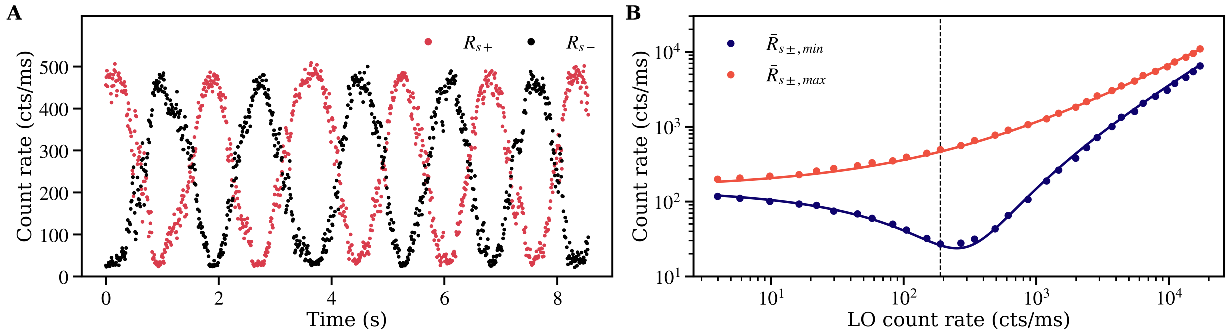

Figure S1: (**A**) Time-traced counts of the two homodyne BS outputs, R_{s+} and R_{s-}, while scanning phase ϕ continuously with a constant excitation power of 2 µW and a LO count rate of 189.1 cts/ms. (**B**) The averaged minima $\bar{R}_{s\pm,\mathrm{min}}$ and maxima $\bar{R}_{s\pm,\mathrm{max}}$ from phase-scanned measurements with varying LO count rate are shown as blue and red symbols. By fitting the theoretical model (solid lines) to the experimental data, the count rates of the mean field $R_{\langle\sigma\rangle} \approx 252.2\,\mathrm{cts/ms}$ and fluctuations $R_{\varsigma} \approx 47.5\,\mathrm{cts/ms}$ as well as the driving $\Omega \approx 0.15$ are obtained. The dashed line represents the LO intensity, where the results in panel **A** are obtained.

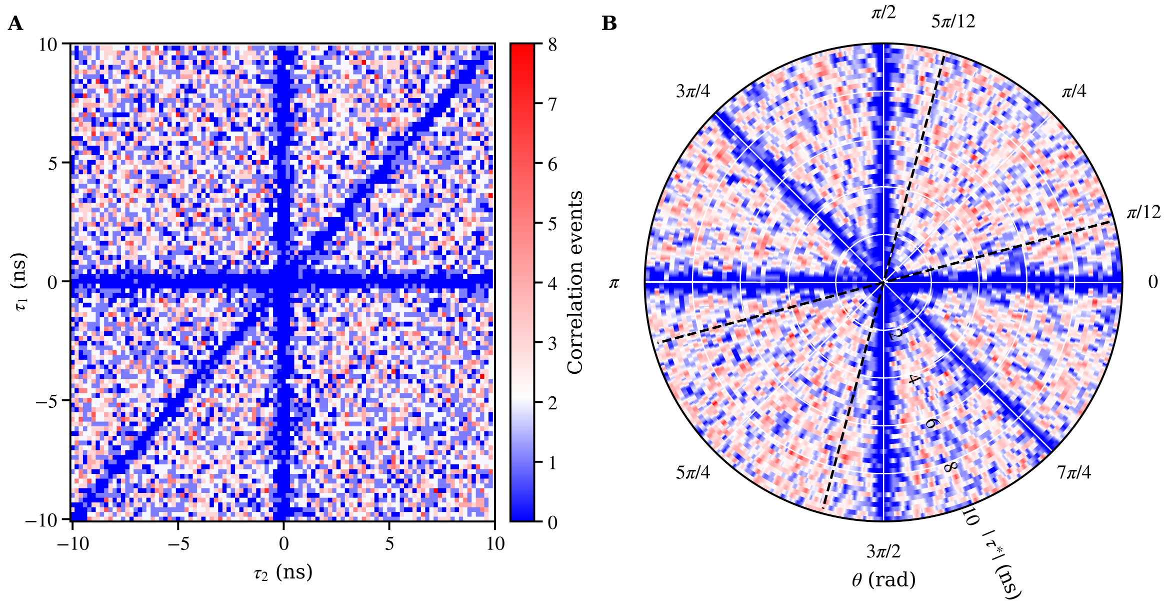

Figure S2: (**A**) With time difference of three detector channels, unnormalized third-order correlation $G^{(3)}(\tau_1, \tau_2)$ is obtained in the Cartesian coordinate system under the measurement condition of $\Omega \approx 0.15$ and $\mathscr{F} = 0$. (**B**) The result is transformed in a polar coordinate system as $G^{(3)}(\tau^*, \theta)$. We perform the radial integration along the θ axis with the integration window centered $\phi = \pi/4$. Dashed lines represent the limit of integration angles $(\pi/12)$ and $(\pi/2 - \pi/12)$.

Table S1: **Summarized correlation measurement data for $\Omega \approx 0.15$.** Shown are the unnormalized second-order correlations at zero time delay, $G^{(2)}(0)$, and at large time delay, $\bar{G}^{(2)}_{\infty}$, as well as the radially integrated third-order correlations at zero delay, $G^{(3)}(0)$, and at large delay, $\bar{G}^{(3)}_{\infty}$. Also listed are the measurement time and the mean and standard deviation (std.) of the interference visibility. For $\mathscr{F} = 0$, the visibility is undefined due to the absence of the LO.

$\mathscr{F}$ (a.u.)	$G^{(2)}(0)$ (events)	$\bar{G}^{(2)}_{\infty}$ (events)	$G^{(3)}(0)$ (events)	$\bar{G}^{(3)}_{\infty}$ (events)	Measurement time (hours)	Mean of visibility (a.u.)	Std. of visibility (a.u.)
6.00	48363	50255.1	175	180.92	0.92	0.30	0.04
5.60	53474	55731.6	157	165.66	1.50	0.32	0.04
5.03	31063	32551.6	56	64.31	1.91	0.33	0.05
4.17	99090	104215.0	76	96.48	26.06	0.48	0.05
3.38	148583	155296.6	182	142.35	46.88	0.44	0.07
2.94	245986	248526.7	291	248.11	56.31	0.56	0.05
2.67	2984	2699.3	5	1.59	2.58	0.55	0.07
2.18	1960	1365.4	7	0.46	4.04	0.65	0.09
1.96	1655	1005.4	3	0.28	5.30	0.69	0.06
1.48	2748	612.2	3	0.12	13.43	0.80	0.08
1.10	5506	433.4	4	0.05	37.96	0.87	0.07
0.42	2944	1133.9	0	0.08	69.92	0.66	0.06
0.00	43	7132.7	0	2.02	19.19	-	-

Table S2: **Summarized correlation measurement data for $\Omega \approx 0.28$.** Shown are the unnormalized second-order correlations at zero time delay, $G^{(2)}(0)$, and at large time delay, $\bar{G}^{(2)}_{\infty}$, as well as the radially integrated third-order correlations at zero delay, $G^{(3)}(0)$, and at large delay, $\bar{G}^{(3)}_{\infty}$. Also listed are the measurement time and the mean and standard deviation (std.) of the interference visibility. For $\mathscr{F} = 0$, the visibility is undefined due to the absence of the LO.

$\mathscr{F}$ (a.u.)	$G^{(2)}(0)$ (events)	$\bar{G}^{(2)}_{\infty}$ (events)	$G^{(3)}(0)$ (events)	$\bar{G}^{(3)}_{\infty}$ (events)	Measurement time (hours)	Mean of visibility (a.u.)	Std. of visibility (a.u.)
6.22	56777	56931.7	917	846.21	0.05	0.21	0.04
5.60	110810	111037.6	1198	1154.40	0.20	0.27	0.04
4.97	21951	21815.3	169	137.83	0.11	0.36	0.04
4.37	12837	12690.6	98	55.86	0.13	0.40	0.04
3.67	7621	7029.0	40	22.62	0.01	0.48	0.03
3.04	3162	2462.3	17	5.39	0.12	0.55	0.07
2.47	3417	1797.6	10	2.17	0.31	0.64	0.06
1.88	6795	1533.5	11	1.02	1.28	0.76	0.06
1.24	14665	1920.7	19	0.89	4.30	0.76	0.08
0.61	17484	7038.6	4	2.49	13.60	0.63	0.05
0.15	295	4938.5	0	2.87	2.91	0.21	0.02
0.00	60	9365.2	0	16.00	2.20	-	-

Table S3: **Summarized correlation measurement data for $\Omega \approx 0.40$.** Shown are the unnormalized second-order correlations at zero time delay, $G^{(2)}(0)$, and at large time delay, $\bar{G}^{(2)}_{\infty}$, as well as the radially integrated third-order correlations at zero delay, $G^{(3)}(0)$, and at large delay, $\bar{G}^{(3)}_{\infty}$. Also listed are the measurement time and the mean and standard deviation (std.) of the interference visibility. For $\mathscr{F} = 0$, the visibility is undefined due to the absence of the LO.

$\mathscr{F}$ (a.u.)	$G^{(2)}(0)$ (events)	$\bar{G}^{(2)}_{\infty}$ (events)	$G^{(3)}(0)$ (events)	$\bar{G}^{(3)}_{\infty}$ (events)	Measurement time (hours)	Mean of visibility (a.u.)	Std. of visibility (a.u.)
4.39	13384	11342.3	170	107.08	0.03	0.26	0.05
3.84	7511	5772.1	79	34.99	0.03	0.34	0.05
3.25	4652	2956.2	50	11.98	0.04	0.42	0.06
2.77	6156	2961.1	50	7.97	0.09	0.50	0.06
2.22	11186	3705.7	59	6.68	0.29	0.57	0.07
1.66	13114	2883.3	37	3.14	0.63	0.67	0.07
1.10	21899	5335.4	29	4.06	0.93	0.64	0.05
0.54	8489	9138.3	7	7.71	0.86	0.43	0.03
0.19	512	7448.9	0	8.39	0.84	0.13	0.02
0.00	135	23986.7	0	63.24	0.77	-	-
